# The Engineering Side of Hospital Work

**Published:** 1905-04-08

**Authors:** 


### HOSPITAL ADMINISTRATION.

### CONSTRUCTION AND ECONOMICS.

### THE ENGINEERING SIDE OF HOSPITAL WORK.

VIII.—LAUNDRIES.

(*Continued from page* 16.)

Among the apparatus auxiliary to the washing machine are the soap and soda dissolver. This is another cylinder, usually fixed vertically, and made of galvanised iron, having a copper pipe, in the form of a coil, in the bottom, and a cock to draw off the solution. It will easily be understood that it would be very wasteful simply to cut up the soap, and put it in the machine, and to put in the soda in lumps. Solution takes time, and goes on better with soap and soda when heat is present. Where soap is simply put into the machine in pieces some of the clothes in the immediate neighbourhood of the individual pieces will get more than they require, while those at a distance may get very little. The operation of heating the water in which the soap and soda are to be dissolved aids the solution by creating a circulation of the water, the water that has been heated rising, and the colder falling, and so on. Another useful ally is a boiling trough, corresponding to the copper of the old days. It is a galvanised tank, fitted with a copper coil at the bottom, through which steam can be passed. The water softener, which was explained in the columns of The Hospital some months since, is another useful agent. In the laundry it saves soap and soda. The salts, which cause what is known as hardness in water, use up soap and soda in the process of neutralising them before the operation of washing can be properly carried out. Practical washermen find it necessary to keep enough soap liquor in the washing machine to keep a good lather all the time the clothes are subject to its action, otherwise it is stated that the impurities of the water sometimes combine with the fatty matters of the soap and leave small particles on the linen that are very difficult to remove. This means that plenty of soap must be dissolved.

Another form of washing machine made by Messrs. Manlove, Alliott and Company is the "Macalpine," in which the "dolly," so much used in the North and the Midlands, is employed. The "dolly," as it is called in the Midlands (the "poss" as it is called farther north), is very much like a small wooden stool with a long handle. It is used in connection with a tub having the form of a cask with the head out. The washer, having put the clothes in the tub together with water and soap, beats the clothes by continually pushing the "dolly" into them till the dirt is all loosened. In the "Macalpine" machine there is a large iron tub cylindrical in form, open at the top, with a beam crossing the top some distance above. To the beam are fixed a number of "dollies" of a particular shape, arranged like the stamp-mill of a metalliferous mine, and worked in the same way. Driving gear is attached to the machine, causing each of the dollies in turn to rise and then fall, the tub being caused to revolve at the same time. Water that has been supplied with soap and soda is placed in the tub, and steam is admitted, the solution being kept at boiling point the whole time the clothes are in the tub. The tub is fitted with a perforated false bottom, the steam-pipes being underneath it, and the dirt and other matters that are removed by the "dollies," and the solvent action of the soap and soda, pass down through the perforations into the false bottom, keeping clear of the clothes in the tub. The "dollies" are made of oak or lime-wood. The rising and falling of the "dollies" is accomplished in a very simple manner. Each "dolly" has a lug on one side, which engages with a double crescent-shaped piece carried by a shaft and revolved by power. As one portion of the crescent-shaped piece comes under the lug of the "dolly" it controls, it raises it, and at another point it suddenly releases the lug, the "dolly" falling on to the clothes below it. This is the action of the stamp mill. It is claimed that with this apparatus the clothes are always in sight and easily accessible, and that considerable economies are realised by its use. It is also claimed that the machine is adapted for all kinds of goods, from canvas tents to lace, and that the machine has stood the test of years.

Another apparatus, made by Messrs. Manlove, Alliott and Co., designed especially for washing blankets, is constructed as follows:—There is a large tub, usually rectangular, open at the top, and having inside two corrugated boards, one of which is movable, and the other stationary, except that it is arranged to give slightly, by mechanical connection with some rubber springs provided for the purpose. The clothes to be washed are folded up concertina fashion (the makers instruct users), and placed between the two boards, water and soap and soda being put in the tub in the usual way. The moving board is worked to and fro by power, squeezing the clothes between the two boards as it advances, and also, by reason of the special character of its motion, slightly rubbing the clothes up against the corrugations of the boards. As the moving board recedes, the clothes, being released, and subjected to the pressure of the water, roll over, presenting a new set of surfaces to the next advance of the moving board. In this way successive surfaces of the clothes are presented to the rubbing surfaces. It is claimed that by this arrangement the clothes are subjected to the minimum of wear, and that the dirtiest clothes can be washed. There are numerous other accessories, one of which is a soap-cutting machine, made by Messrs. Summerscales. Soap dissolves more readily if cut up in thin slices, and the machine is arranged to take a bar of soap of any size, and, by successive depressions of a lever, to slice off pieces as thick as the laundry manager desires. The apparatus is fixed on the end of a table in any convenient position, the lever being worked by hand.

In the next article the writer will deal with arrangements for drying the clothes.

